# Quenching ilmenite with a high-temperature and high-pressure phase using super-high-energy ball milling

**DOI:** 10.1038/srep04700

**Published:** 2014-04-25

**Authors:** Takeshi Hashishin, Zhenquan Tan, Kazuhiro Yamamoto, Nan Qiu, Jungeum Kim, Chiya Numako, Takashi Naka, Jean Christophe Valmalette, Satoshi Ohara

**Affiliations:** 1 Joining and Welding Research Institute, Osaka University, 11-1, Mihogaoka, Ibaraki, Osaka 567-0047, Japan; 2 SPring-8/Japan Synchrotron Radiation Research Institute, 1-1-1 Kouto, Sayo-cho, Sayo-gun, Hyogo 679-5198; 3 Graduate School of Science, Chiba University, 1-33, Yayoi-cho, Inage-ku, Chiba-shi, Chiba 263-8522, Japan; 4 Fine Particles Engineering Group, Advanced Materials Processing Unit, National Institute for Materials Science, 1-2-1, Sengen, Tsukuba, Ibaraki 305-0047, Japan; 5 IM2NP UMR 7334 CNRS, Université du Sud Toulon Var, P.O. Box 20132, 83957 La Garde CEDEX, France

## Abstract

The mass production of highly dense oxides with high-temperature and high-pressure phases allows us to discover functional properties that have never been developed. To date, the quenching of highly dense materials at the gramme-level at ambient atmosphere has never been achieved. Here, we provide evidence of the formation of orthorhombic Fe_2_TiO_4_ from trigonal FeTiO_3_ as a result of the high-temperature (>1250 K) and high-pressure (>23 GPa) condition induced by the high collision energy of 150 gravity generated between steel balls. Ilmenite was steeply quenched by the surrounding atmosphere, when iron-rich ilmenite (Fe_2_TiO_4_) with a high-temperature and high-pressure phase was formed by planetary collisions and was released from the collision points between the balls. Our finding allows us to infer that such intense planetary collisions induced by high-energy ball milling contribute to the mass production of a high-temperature and high-pressure phase.

Silica^1^,^2^ and titania^3^ with α-PbO_2_-like structures are found in the Shergotty meteorite^1^,^2^ and Ries crater^3^. It has been demonstrated that these oxides were formed at high pressures of 70^4^ and 20 GPa^5^, respectively, by shock events on the meteorite and in Ries crater. Spectroscopic measurements achieved through in situ diamond anvil cell, shock wave, and laser-driven experiments have been used as artificial methods to study the phase transitions of the materials under high pressure. Theoretical calculations based on the in situ diamond anvil cell experiments revealed that SiO_2_ undergoes several phase transitions to post-stishovite polymorphs above 48 GPa^9^,^10^,^11^. Shock wave experiments on rutile at peak pressures of 20 GPa revealed a phase transformation to an α-PbO_2_-type polymorph^12^,^13^,^14^,^15^. Synthetic vitreous silica transforms partially into a defective form of a high-pressure stishovite phase under high-intensity laser irradiation^16^. In shock-induced synthesis, planetary ball milling is generally used to mix the starting materials and alloying metals^17^, and it has been estimated to use several hundred kilojoules for a milling time of several hundred hours^18^. If highly dense materials were formed under high-temperature and high-pressure conditions generated by the collision energy of planetary ball milling, their unique functional properties could be determined.

The Allende meteorite and the Martian meteorite Allan Hills (ALH) 84001 host graphitic nanocarbons^6^,^7^, which are formed by induced shock events between 2–6 GPa^8^. In a previous study, we reported a simple shock event approach that was inspired by the Allende meteorite to produce sophisticated carbon nanomaterials including carbon nanotubes, carbon onions, and carbon nanorings^19^,^20^. These materials were synthesised by a collision shock between steel balls using a super high-speed ball-milling process. When synthesising oxides using planetary ball milling, it is known that the anatase-rutile phase transformation of titania can occur only when the anatase cluster reaches a critical size of 10–15 nm^21^,^22^,^23^,^24^. However, there have been no reports to date of forming iron-titanate using a planetary ball-milling process using titania powder as the starting material and a steel ball and vial as the collision medium. Thus, the collision energy of the conventional planetary ball-milling process is too low to form ilmenite. However, we succeeded in forming ilmenite using super-high-energy ball milling between anatase nano-powders and steel balls^25^. Generally, ilmenite is formed by a solid-state reaction at a temperature exceeding 1200°C^26^. The results suggested that the temperature inside the stainless steel vial increased to greater than 1200°C due to the high impact energy of the collision shock between the balls.

Recently, the phase transition of ilmenite from trigonal to orthorhombic inside a diamond anvil cell was demonstrated at high temperature (<2500 K) and high pressure (<75 GPa) using in situ X-ray diffraction with synchrotron radiation^27^. In this case, the ilmenite phase returned to the trigonal phase under ambient temperature and pressure. In addition, ilmenite with a high-temperature and high-pressure phase has been shown to become unquenchable at ambient conditions^28^. This study was the first to report quenching ilmenite with a high-temperature and high-pressure phase using super-high-energy ball milling. When ilmenite with a high-temperature and high-pressure phase is formed by collision shock and is released from the collision points between the balls, it would be expected to be steeply quenched by the surrounding atmosphere. We succeeded in obtaining results that demonstrate the existence of ilmenite with high-temperature and high-pressure phases. These results were supported by analytical measurements based on X-ray diffraction with synchrotron radiation and ultrahigh-resolution transmission electron microscopy.

## Results

**Figure 1:**
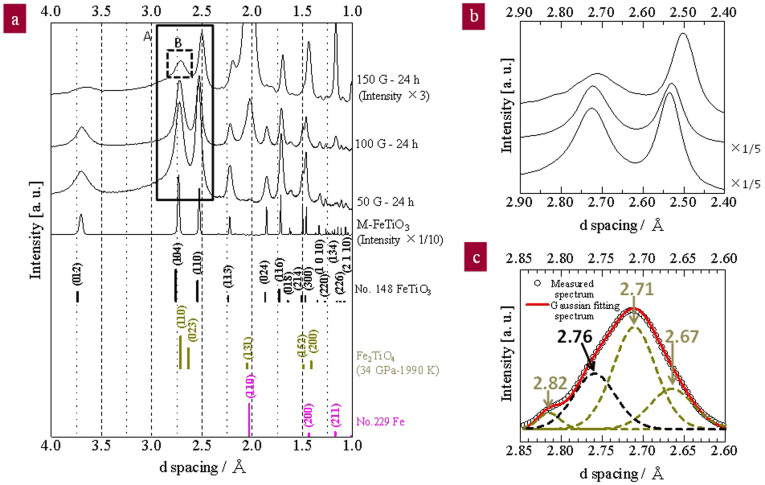
XRD patterns of iron titanate as milled at 50, 100, and 150 G for 24 h. (a) Product phase as-milled by collision energies of 50, 100, and 150 G for 24 h. The bar graphs are based on the database of XRD patterns: the space number is extracted from the NIMS atom work and Fe_2_TiO_4_ from ref. 27. (b) An enlarged view of solid square A. (c) Gaussian deconvolution of dotted square B. The adjusted R-square is 0.999.

The raw material, labelled as M-FeTiO_3_, was milled at a high-energy collision of 50, 100, and 150 G for 24 h (see the details of the experimental setup in the Methods section). These samples were then characterised using X-ray diffraction spectroscopy (XRD), as shown in Fig. 1. The peak position of the raw powder was almost shifted to a lower d-spacing of trigonal FeTiO_3_. The relative peak intensity was drastically decreased and the full width at half maximum, FWHM, was remarkably increased by the collision shock of 50 G, suggesting a downsizing at the nano-level by the collision shock between the steel balls and the inner wall of the vial. The sample as-milled at 100 G for 24 h exhibited a slight decrease in the relative peak intensity and in the d-spacing of trigonal FeTiO_3_ and a remarkable increase in iron content (Fig. 1a).

Notably, the peak position of the as-milled sample was considerably shifted in the decreasing direction of the d-spacing with increasing collision energy from 100 to 150 G (Fig. 1b). The lattice of the trigonal FeTiO_3_ could be compressed by the collision shock between steel balls and the inner wall of vial, leading to the formation of orthorhombic Fe_2_TiO_4_. The Gaussian deconvolution of the dotted square B indicates the best agreement with the third peak from the first peak of orthorhombic Fe_2_TiO_4_ as well as the first peak of trigonal FeTiO_3_ (Fig. 1c).

**Figure 2:**
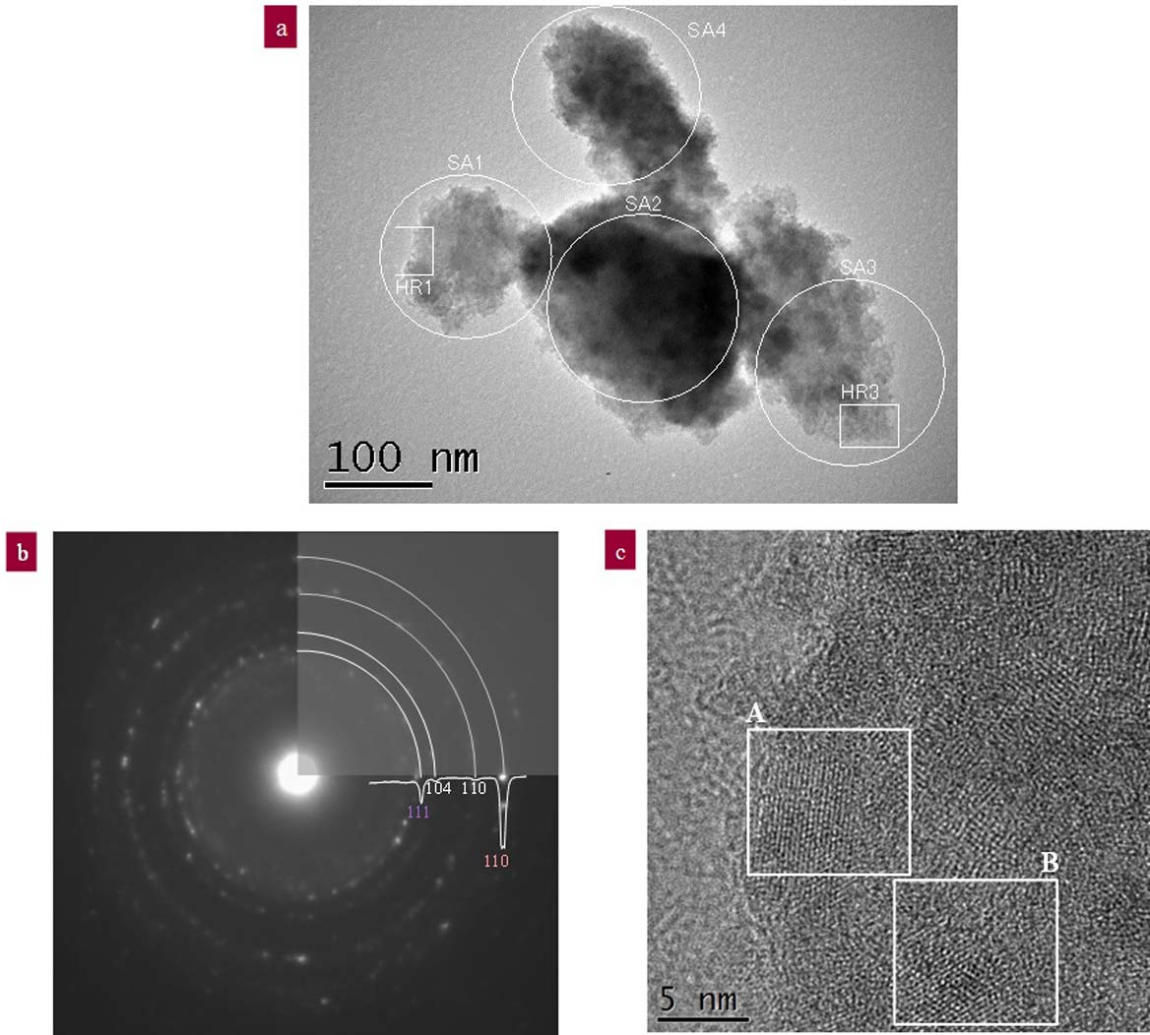
Morphological and structural features of iron titanate as-milled at 150 G for 24 h. (a) TEM image of four selected areas (SA): SA1, SA2, SA3, and SA4. HR1 and HR3 are the high-resolution images of SA1 and SA3, respectively. (b) Ring diffraction patterns of SA1 in Fig. 2a. The top-right corner is the result of circular averaging of the pattern. A lineout through the scanned diffraction pattern is included. The ring diffraction patterns of SA2–4 in Fig. 2a are summarised in Fig. S2a–c. (c) High-resolution TEM images of areas A and B. The d-spacings of both areas are summarised in Table 2.

The morphological and structural features of the iron titanate as milled at 150 G for 24 h are shown in Fig. 2. The diameter of the iron titanate nanoparticles ranged from 5 to 20 nm, and their aggregates were between 100 to 200 nm in size. We investigated the electron diffraction patterns of four circular areas and the high-resolution images of two square areas to identify the product phases. A representative sample was then selected to obtain an area electron diffraction image of SA1 and a high-resolution image of HR1, as presented in Figs. 2b and 2c, respectively. The electron diffraction patterns from SA1 in Fig. 2a indicate the presence of small crystallites.

We compared the experimental diffraction patterns to all of the known high-pressure FeTiO_3_ polymorphs (trigonal, perovskite, orthorhombic Fe_2_TiO_4_, orthorhombic intermediate TiO_2_, and wüstite)^27^. The best agreement of all the FeTiO_3_ polymorphs was trigonal FeTiO_3_, OI-TiO_2_ (Table 1). The very strong diffraction of Fe (110) appeared as a first-order diffraction in Fig. 2b. In the supplementary Table S1c, the presence of Fe (110) is also observed as a diffraction peak. The formation amount of Fe is quite large after being milled at 150 G for 24 h. The second strongest reflection in Fig. 2b was assigned to OI-TiO_2_. Hamane et al. clarified that OI-TiO_2_ coexists with Fe_2_TiO_4_^27^.

**Figure 3:**
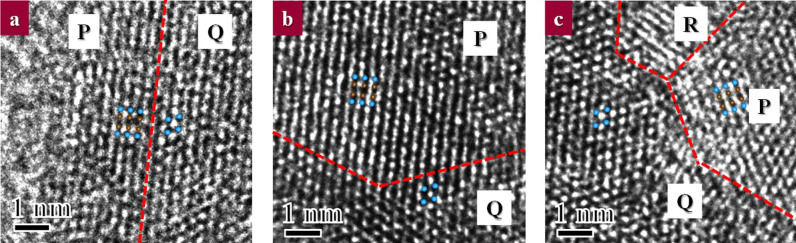
High-resolution TEM images of ilmenite as-milled at 150 G for 24 h. The periodic atom arrangements are confirmed in three typical images of (a), (b), and (c). Regions P, Q, and R correspond to orthorhombic ilmenite (Fe_2_TiO_4_), orthorhombic intermediate titania (OI-TiO_2_), and trigonal ilmenite (FeTiO_3_), respectively. Dotted line on each image shows the boundaries of each region, and brown ball is Fe atom and light blue ball Ti atom.

The presence of OI-TiO_2_ and Fe_2_TiO_4_ indicates that the condition of high temperature and high pressure could be temporally produced by the collision shock between the steel balls and vial. The interplanar spacings measured from the high-resolution images of areas A and B indicate the best agreement with the d-spacing of Fe_2_TiO_4_ (110), corresponding to the maximum relative peak intensity. The other interplanar spacings indicate good agreement with the d-spacing of OI-TiO_2_ and FeTiO_3_ in Table 2. Partially magnified images of the square area A and the another two areas are presented in Fig. 3 to confirm the periodicity of the interplanar spacing. Three product phases were confirmed from the ultra-high-resolution images of the three areas, Figs. 3 a, b, c. Regions P, Q, and R show good agreement with iron-rich ilmenite (Fe_2_TiO_4_), orthorhombic intermediate titania (OI-TiO_2_), and trigonal ilmenite (FeTiO_3_), respectively. The d-spacings of two or three regions in each figure are listed in Table 3. The lattice fringes of region P with a d-spacing of 2.73 nm on average were much closer to the (110) lattice fringes of Fe_2_TiO_4_ with a d-spacing of 2.71 nm. In addition, the lattice fringes of region Q with a d-spacing of 3.13 nm on average were much closer to the (111) lattice fringes of OI-TiO_2_ with a d-spacing of 3.13 nm. The lattice fringes of Fe_2_TiO_4_ appeared as a third peak as well as first and second peaks. This result is in good agreement with the three peak positions of Fe_2_TiO_4_ determined through the Gaussian deconvolution of the XRD profile of Fig. 1c.

## Discussion

The peak intensity of iron was relatively increased with increasing collision energy. The iron was considered to be generated from both the surface of the steel balls and the inside wall of the steel vial. Iron oxides such as Fe_2_O_3_ and Fe_3_O_4_ did not appear as a new peak, which indicates that the reduction atmosphere could be maintained inside the vial. The existence of iron most likely contributes to the formation of an iron-rich ilmenite phase. The iron and oxygen within the vial might be partially incorporated as FeO in the lattice of FeTiO_3_ to form Fe_2_TiO_4_. However, a detailed investigation is required to clarify the actual formation mechanism of Fe_2_TiO_4_. An experiment on an in situ diamond anvil cell confirmed the coexistence of Fe_2_TiO_4_, intermediate orthorhombic (OI)-TiO_2_, and FeO at high temperatures exceeding 1250 K and high pressures ranging from 23 to 37 GPa^27^. Orthorhombic Fe_2_TiO_4_ with a high-temperature and high-pressure phase could be formed from a super high-energy collision shock of 150 G. This outcome can be presumed from the appearance of the first and second peaks in the Fe_2_TiO_4_ spectra, as observed in Fig. 1c. In particular, the low index plane of the third peaks of Fe_2_TiO_4_ (200), i.e., Fe_2_TiO_4_ (100), was confirmed at the d-spacing of 2.82 Å by the Gaussian deconvolution. The existence of this peak is clear compared with the peak with the d-spacing of 2.67 Å and strongly supports the existence of periodic atoms arrangements of ilmenite as-milled at 150 G for 24 h.

Laser-induced damage within the composites grows catastrophically once initiated, thereby dramatically shortening the potential exposure time of these materials under high-powered photonic devices. Accordingly, the existence of copper in silica contributes to thermal quenching, which causes the high-pressure phase transition at ambient conditions^29^,^30^,^31^. The thermal conductivity of iron, 28.0 W/m·K^32^, is much higher than that of iron titanate, 1.82 W/m·K^33^. This clear difference in the conductivity suggests that iron plays the role of quenching the iron-rich ilmenite with a high-temperature and high-pressure phase.

**Figure 4:**
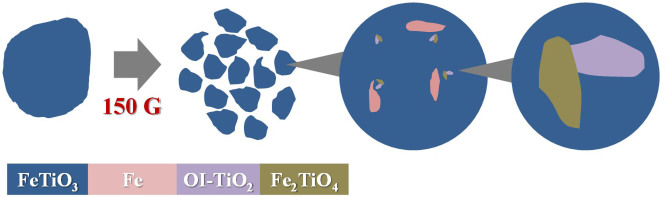
Formation mechanism of the high-temperature and high-pressure phase.

Based on the above discussion, the formation mechanism of the high-temperature and high-pressure phase is illustrated in Fig. 4. Initially, friction heat was generated by the shear stress of the collision shock between the steel balls and vial. FeTiO_3_ was continuously ground by the collision shock. The small amount of Fe_2_TiO_4_ could be formed between Fe and FeTiO_3_ near Fe fragments based on the existence of the iron fragments within the Fe-Ti-O compounds clarified by the element mapping of the cross-section of SUS440C shown in Fig. S2.

In summary, 149-μm diameter ilmenite grains were milled at 150 G for 24 h using super high-energy ball milling to quench the high-temperature and high-pressure phase. It was clarified by spectroscopic observations that the product consisted of trigonal FeTiO_3_ as the main phase and orthorhombic Fe_2_TiO_4_ as the high-temperature and high-pressure phase. We believe that super high-energy ball milling would provide us with the possibility of synthesising highly dense materials such as silica and titania under high-temperature and high-pressure conditions, which will uncover undiscovered potential superior to the conventional functionality of nano-oxides and ceramics by enhancing the collision energy.

## Methods

### Experimental setup

The principle and apparatus of super high-energy ball milling is illustrated in Fig. S3a. We can calculate the revolution number (N) of planetary ball milling using the theoretical equation of a motor. When N is substituted with the angular velocity (ω), the equation “ω = F (π/2)” is obtained. The equation is substituted with the theoretical equation of centrifugal force (g*). We can control the value of g* using the equation g* = 0.0441 F^2^.

The evaluation of heat generation from collision energy is shown in Fig. S3b. The collision shock event between steel balls consists of F(normal), which is related to the centrifugal force, and F(shear), which is related to the shear stress between the balls. Based on this concept, the collision energy of planetary ball milling has been reported to be 1.83 J/s·g for F(normal) and 2.54 J/s·g for F(shear) by the simulation. From these values, we calculated the heat generation of one steel ball using a specific heat capacity of 0.458 J/K·g when the vial was assumed to be insulated. As a result, heat of 165 K/ball and 228 K/ball could be generated by F(normal) and F(shear), respectively. When each ball gathers around each other similar to hexagonal close packing, the heat generation will momentarily become 12 times these values. We cannot measure the actual heat generation with collision shock between the steel balls and the vial. From the above consideration, we predict that the condition of high temperature (1990 K) and high pressure (34 GPa) occurred due to the super high-energy ball milling based on the results for Fe_2_TiO_4_.

The raw material, meta-FeTiO_3_, is commercially available and had a mean particle size of 149 μm. First, 10 cm^3^ of FeTiO_3_ powder (M-FeTiO_3_) was loaded into a 170-cm^3^ cylindrical vial together with 50-cm^3^ milling balls. The milling balls were commercial stainless steel balls that consisted of a solid solution of iron, chromium, and carbon with a 3-mm diameter. Mechanochemical treatment was performed using a super high-speed ball-milling apparatus that operated for 24 h in an air atmosphere under various centrifugal forces of 50, 100, and 150 G.

### Characterisation

The samples were filled inside a capillary with an 80-μm inner diameter and were scanned at the BL02B2 beam line (SPring-8) with a wavelength of 0.35441 Å. A series of XRD spectra were collected on an imaging plate using an exposure time of 10 min. A small piece of the produced nanostructures was suspended in ethanol (1.0 mL) by ultrasonication until a homogeneous suspension was obtained. The suspension was dropped onto a carbon-coated Cu grid, dried, and examined by transmission electron microscopy (TEM) operated at 200 kV (JEM-2100F, JEOL, Japan).

## Supplementary Material

Supplementary Information NEW Supplementary Information File

## Figures and Tables

**Table 1:** Interplanar spacing measured from TEM diffraction patterns of selected area 1 in Fig. 2a and spacings of the trigonal FeTiO_3_, orthorhombic intermediate(OI) TiO_2_ and orthorhombic Fe_2_TiO_4_. The spacing |Δd| is the difference between the d-spacing observed and that recorded in the database, I. D. Phase is the product phase identified by considering |Δd| and R. Int. corresponds to the relative peak intensity in the database. The selected areas 2–4 are summarised in Table S1a–c

*d_obs_* (Å)	Apparent Intensity	*d_hkl_* FeTiO_3_ (Å)	*hkl*	|Δd| (%)	*d_hkl_* OI-TiO_2_ (Å)	*hkl*	|Δd| (%)	*d_hkl_* Fe (Å)	*hkl*	|Δd| (%)	*d_hkl_* Fe_2_TiO_4_ (Å)	*hkl*	|Δd|(%)	I. D. Phase (R. Int.)
					3.32	210								
3.13	Strong				**3.15**	**111**	**0.6**							**OI-TiO_2_ (3rd)**
2.82	Weak	**2.76**	**104**	**2.2**							2.71	110	3.9	**FeTiO_3_ (1st)**
											2.63	023		
2.52	Weak	**2.54**	**110**	**0.8**										**FeTiO_3_ (2nd)**
					2.26	102								
					2.15	021								
1.95	Very Strong	1.73	116	12.7	2.10	121	7.1	**2.03**	**110**	**4.0**	**2.05**	**131**	**4.9**	**Fe (1st) Fe_2_TiO_4_ (4th)**

**Table 2:** Interplanar spacings measured from area A and B of high-resolution images and spacings of the trigonal FeTiO_3_, orthorhombic intermediate(OI) TiO_2_ and the orthorhombic Fe_2_TiO_4_. The spacing |Δd| is the difference between the d-spacing observed and that recorded in the database, I. D. Phase is the product phase identified by considering the |Δd| value, and R. Int. corresponds to the relative peak intensity in the database

*d_obs_*(Å)	*d_hkl_*FeTiO_3 _(Å)	*hkl*	|Δd| (%)	*d_hkl_*OI-TiO_2_ (Å)	*hkl*	|Δd| (%)	*d_hkl_*Fe_2_TiO_4_ (Å)	*hkl*	|Δd| (%)	I. D. Phase (R. Int.)
3.71	**3.74**	**012**	**0.8**	3.32	210	11.7				**FeTiO_3_ (4th)**
3.13				**3.15**	**111**	**0.6**				**OI-TiO_2_ (3rd)**
2.73	2.76	104	1.1				**2.71**	**110**	**0.7**	**Fe_2_TiO_4_ (1st)**

**Table 3:** Interplanar spacings measured from regions P, Q, and R of Figs. 3 (a), (b) and (c), and spacings of the trigonal FeTiO_3_, orthorhombic intermediate (OI) TiO_2_ and orthorhombic Fe_2_TiO_4_. The spacing |Δd| is the difference between the d-spacing observed and that recorded in the database, I. D. Phase is the product phase identified by considering the |Δd| value, and R. Int. corresponds to the relative peak intensity in the database

(a)											
Region	*d_obs_*(Å)	*d_hkl_*FeTiO_3 _(Å)	*hkl*	|Δd| (%)	*d_hkl_*OI-TiO_2_ (Å)	*hkl*	|Δd| (%)	*d_hkl_*Fe_2_TiO_4_ (Å)	*hkl*	|Δd| (%)	I. D. Phase (R. Int.)
Q	3.13				**3.15**	**111**	**0.6**				**OI-TiO_2_ (3rd)**
P	2.73	2.76	104	1.1				**2.71**	**110**	**0.7**	**Fe_2_TiO_4_ (1st)**

(b)											
Region	*d_obs_*(Å)	*d_hkl_*FeTiO_3 _(Å)	*hkl*	|Δd| (%)	*d_hkl_*OI-TiO_2_ (Å)	*hkl*	|Δd| (%)	*d_hkl_*Fe_2_TiO_4_ (Å)	*hkl*	|Δd| (%)	I. D. Phase (R. Int.)
Q	3.16				**3.15**	**111**	**0.2**				**OI-TiO_2_ (3rd)**
P	2.86	2.76	104	3.6				**2.82**	**100**	**1.2**	**Fe_2_TiO_4_ (3rd′)**

(c)											
Region	*d_obs_* (Å)	*d_hkl_*FeTiO_3_ (Å)	*hkl*	|Δd| (%)	*d_hkl_*OI-TiO_2_ (Å)	*hkl*	|Δd| (%)	*d_hkl_*Fe_2_TiO_4_ (Å)	*hkl*	|Δd| (%)	I. D. Phase (R. Int.)
Q	3.14				**3.15**	**111**	**0.3**				**OI-TiO_2_ (3rd)**
P	2.70	2.76	104	2.2				**2.71**	**110**	**0.3**	**Fe_2_TiO_4_ (1st)**
P	2.64							**2.63**	**023**	**0.5**	**Fe_2_TiO_4_ (2nd)**
R	2.48	**2.54**	**110**	**2.4**							**FeTiO_3_ (2nd)**
