# Supplementary material for: Quenching ilmenite with a high-temperature and high-pressure phase using super-high-energy ball milling
Source: Sci Rep. 2014 Apr 25;4:4700. doi: 10.1038/srep04700 (PMC5381190; doi:10.1038/srep04700)
Supplement: Supplementary Information — NEW Supplementary Information File [file srep04700-s1.pdf]

Supplementary information

Quenching ilmenite with a high-temperature and high-pressure phase using super-high-energy ball milling

TAKESHI HASHISHIN[1,*], ZHENQUAN TAN[1], KAZUHIRO YAMAMOTO[1], NAN QIU[1], JUNGEUM KIM[2], CHIYA NUMAKO[3], TAKASHI NAKA[4], JEAN CHRISTOPHE VALMALETTE[5], SATOSHI OHARA[1,*]

[1] Joining and Welding Research Institute, Osaka University, 11-1, Mihogaoka, Ibaraki, Osaka 567-0047, Japan

[2] SPring-8 / Japan Synchrotron Radiation Research Institute, 1-1-1 Kouto, Sayo-cho, Sayo-gun, Hyogo 679-5198

[3] Graduate School of Science, Chiba University, 1-33, Yayoi-cho, Inage-ku, Chiba-shi, Chiba 263-8522, Japan

[4] Fine Particles Engineering Group, Advanced Materials Processing Unit, National Institute for Materials Science, 1-2-1, Sengen, Tsukuba, Ibaraki 305-0047, Japan

[5] IM2NP UMR 7334 CNRS, Université du Sud Toulon Var, P.O. Box 20132, 83957 La Garde CEDEX, France

*Correspondence to : T. Hashishin, hasisin@jwri.osaka-u.ac.jp; S. Ohara, ohara@jwri.osaka-u.ac.jp

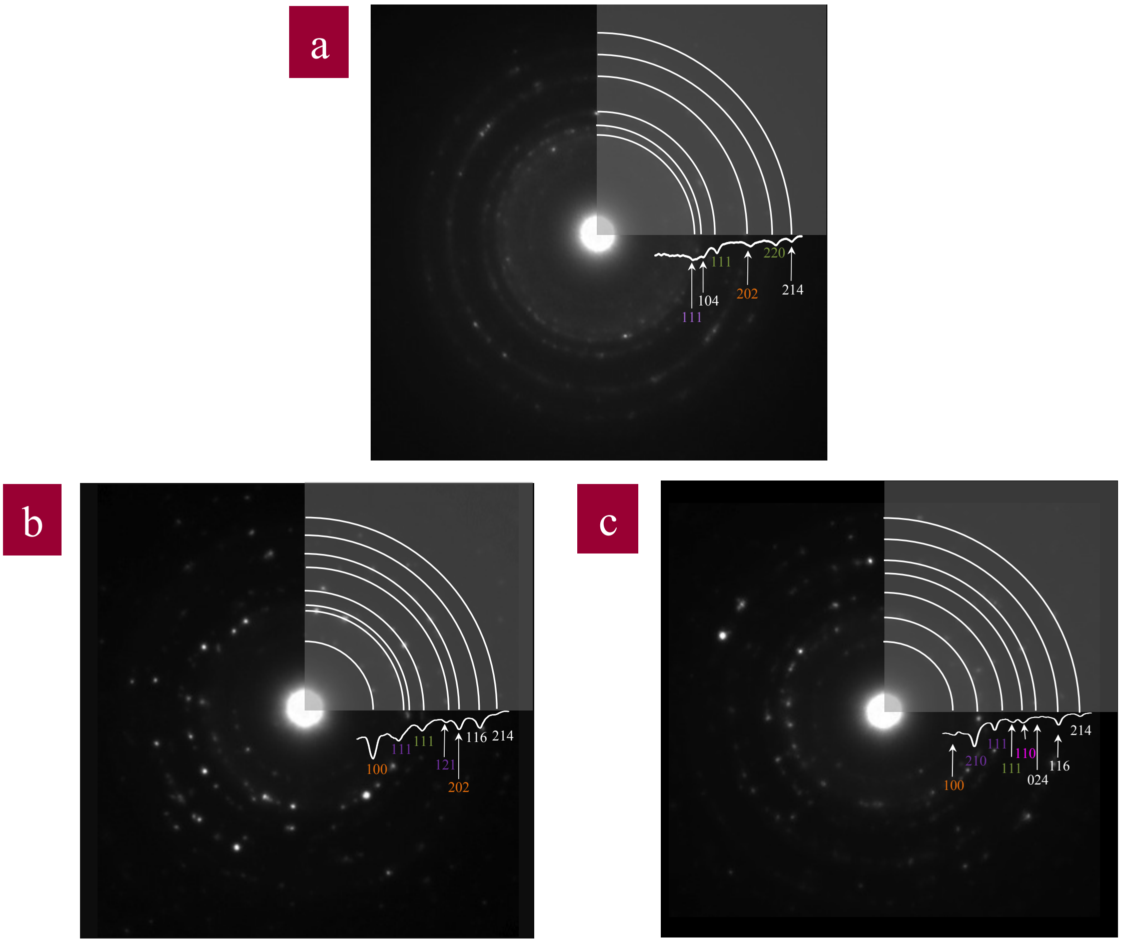

Figure S1 | Ring diffraction patterns of iron titanate as milled at 150 G for 24 h. (a) (b), and (c) are the ring diffraction patterns of SA2, 3, and 4 in Fig. 2a, respectively. The top-right corner is the result of circular averaging of the pattern. A lineout through the scanned diffraction pattern is included.

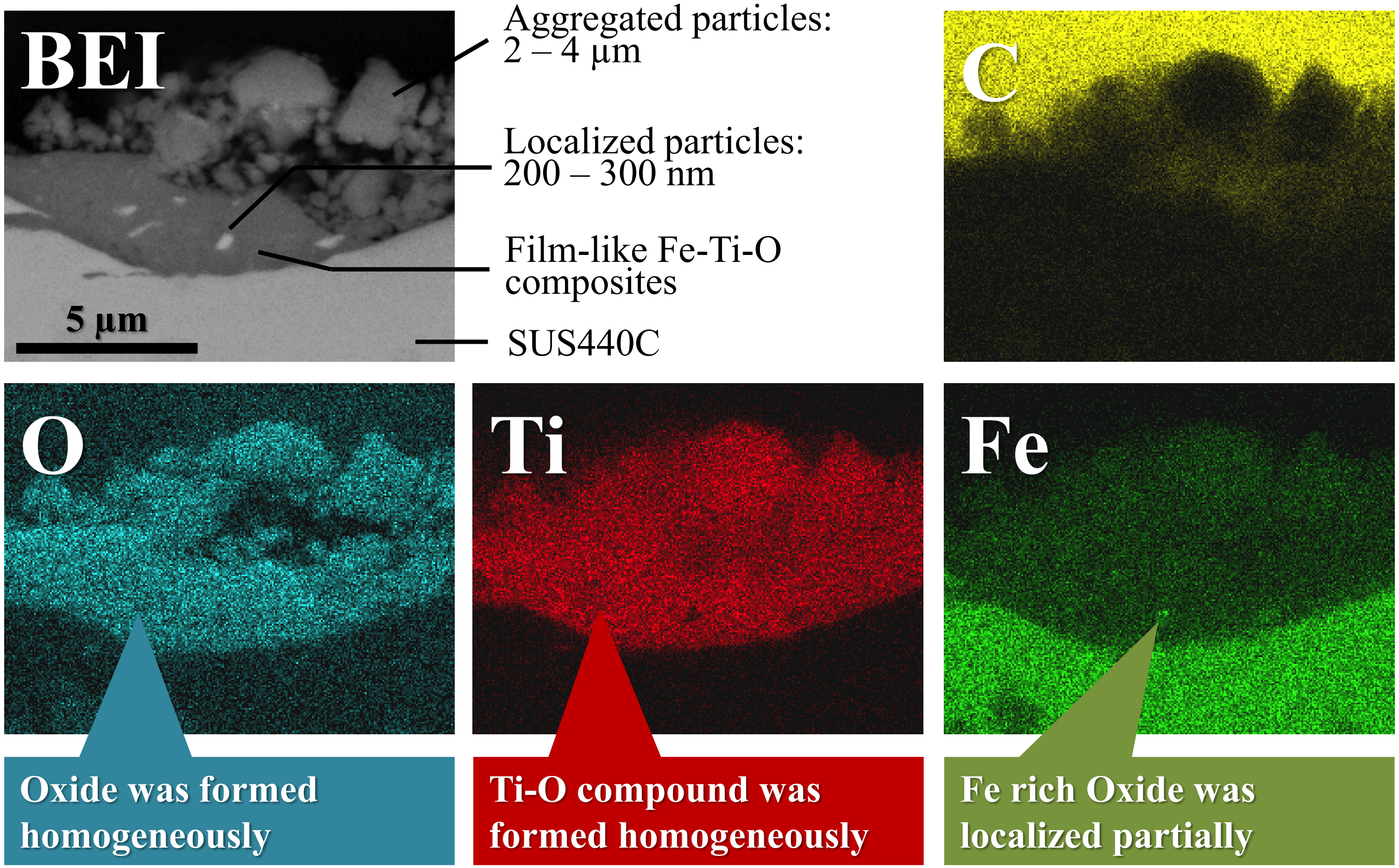

Fig. S2 | Element mapping of cross-section of the SUS440C ball as-milled at 150 G for 10 h.

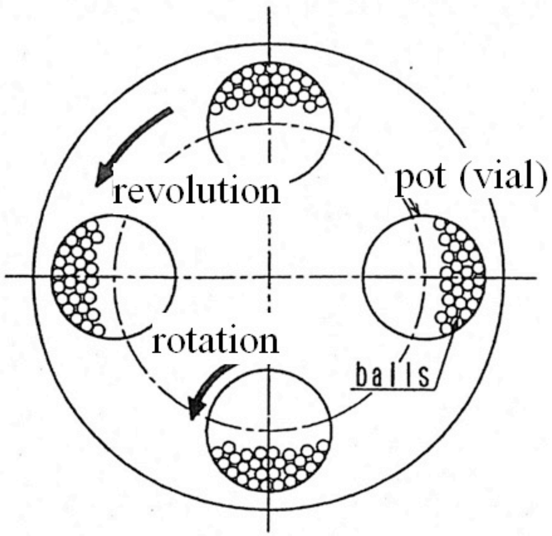

g^*	F/ Hz
150	58.32
100	47.62
50	33.67

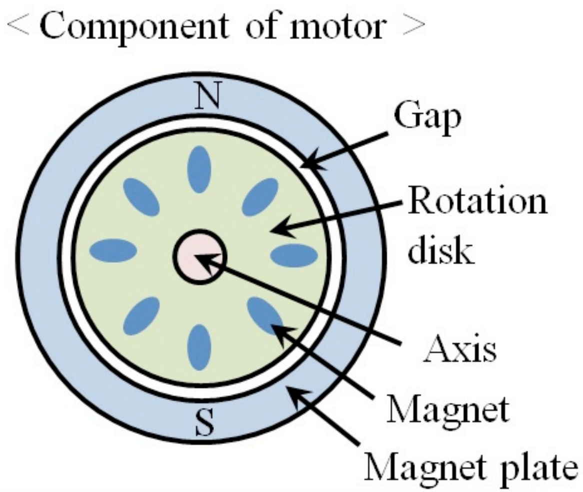

$g^* = 0.0441 \times F^2$

$\omega = F \times \pi/2$

Theoretical equation

N(rpm) = 60 × F(Hz) / (P*/2)

= 2 pole × 4 motors

*:P (pole number)

N(rpm) = 15 × F (Hz)

$g^* = \omega^2 (G + D(1+R)^2) / (2 \times 9.8)$

ω: angular velocity ($2\pi \times N/60$)

G: revolution diameter (0.25 m)

D: inner diameter of pot (0.05 m)

R: ratio of rotation to revolution (=244/583=0.419)

Fig. S3 | (a) Principle and apparatus of super-high-energy ball milling.

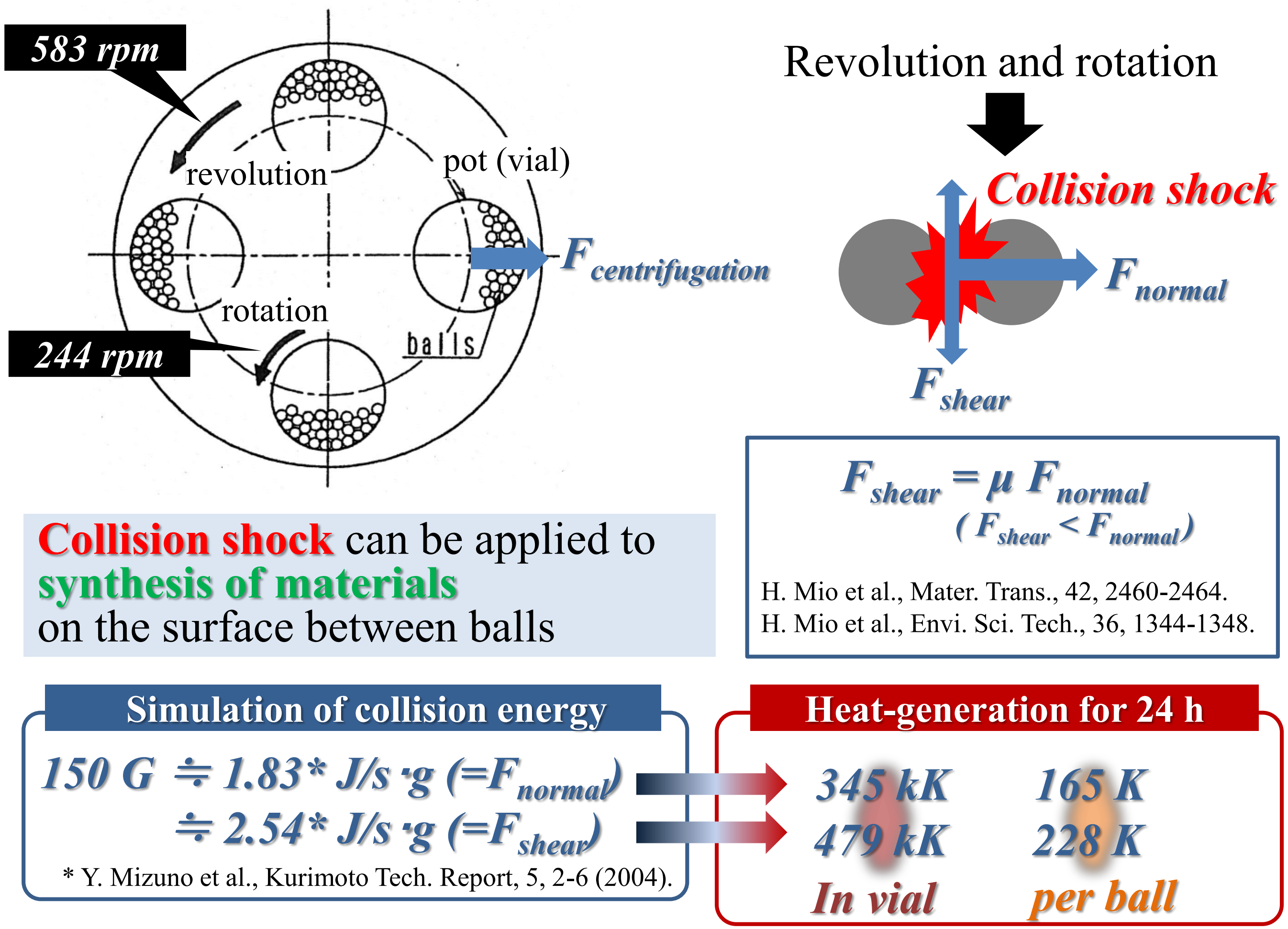

Fig. S3 | (b) Evaluation of heat-generation from collision energy.

Table S1 | Interplanar spacing measured from TEM diffraction patterns and spacings of the trigonal $FeTiO_3$, orthorhombic intermediate(OI) TiO_2, cubic wüstite (FeO), cubic metal (Fe_2Ti) and orthorhombic Fe_2TiO_4. (a), (b), and (c) are the identification lists of SAD2, 3, and 4 in Fig. 2a, respectively. The spacing |Δd| is the difference between the d-spacing observed and that recorded in the database, I. D. Phase is the product phase identified by considering |Δd|, and R. Int. corresponds to the relative peak intensity in the database.

(a)

d_{obs} (Å)	Apparent Intensity	d_{hkl} $FeTiO_3$ (Å)	*hkl*	|Δd| (%)	d_{hkl} OI-TiO_2 (Å)	*hkl*	|Δd| (%)	d_{hkl} FeO (Å)	*hkl*	|Δd| (%)	d_{hkl} Fe_2Ti (Å)	*hkl*	|Δd| (%)	d_{hkl} Fe_2TiO_4 (Å)	*hkl*	|Δd| (%)	I. D. Phase (R. Int.)
					3.32	210					4.14	100					
3.06	Strong				**3.15**	**111**	**2.9**										**OI-TiO_2 (3rd)**
2.77	Strong	**2.76**	**104**	**0.4**										2.71	110	2.2	**$FeTiO_3$ (1st)**
														2.63	023		
2.45	Weak	2.54	110	3.5				**2.49**	**111**	**1.6**							**FeO (1st)**
											2.39	110					
					2.26	102											
											2.20	103					
					2.15	021		2.16	200								
					2.10	121											
											2.04	112		2.05	131		
											2.00	201					
1.89	Weak										**1.83**	**202**	**3.3**				**Fe_2Ti (5th)**
1.60	Weak	1.73	116	7.5				**1.52**	**220**	**5.3**							**FeO (3rd)**
1.47	Weak	**1.51**	**214**	**2.3**													**$FeTiO_3$ (5th)**

(b)

d_{obs} (Å)	Apparent Intensity	d_{hkl} $FeTiO_3$ (Å)	*hkl*	|Δd| (%)	d_{hkl} OI-TiO_2 (Å)	*hkl*	|Δd| (%)	d_{hkl} FeO (Å)	*hkl*	|Δd| (%)	d_{hkl} Fe_2Ti (Å)	*hkl*	|Δd| (%)	d_{hkl} Fe_2TiO_4 (Å)	*hkl*	|Δd| (%)	I. D. Phase (R. Int.)
4.14	Very Strong										**4.14**	**100**	**0.0**				**Fe_2Ti (6th)**
					3.32	210											
3.09	Very Strong	2.76	104	12.0	**3.15**	**111**	**1.9**										**OI-TiO_2 (3rd)**
														2.71	110		
2.49	Strong	2.54	110	2.0				**2.49**	**111**	**0.0**				2.63	023		**FeO (2nd)**
											2.39	110					
					2.26	102											
											2.20	103					
								2.16	200								
					2.15	021					2.04	112					
2.09	Weak				**2.10**	**121**	**0.5**				2.00	201	4.5	2.05	131	2.0	**OI-TiO_2 (2rd)**
1.92	Strong										**1.83**	**202**	**4.9**				**Fe_2Ti (5th)**
1.62	Weak	**1.73**	**116**	**6.4**													**$FeTiO_3$ (3rd)**
1.51	Weak	**1.51**	**214**	**0.3**				1.52	220	0.7							**$FeTiO_3$ (4th)**

(c)

d_{obs} (Å)	Apparent Intensity	d_{hkl} $FeTiO_3$ (Å)	*hkl*	|Δd| (%)	d_{hkl} OI-TiO_2 (Å)	*hkl*	|Δd| (%)	d_{hkl} FeO (Å)	*hkl*	|Δd| (%)	d_{hkl} Fe_2Ti (Å)	*hkl*	|Δd| (%)	d_{hkl} Fe (Å)	*hkl*	|Δd| (%)	d_{hkl} Fe_2TiO_4 (Å)	*hkl*	|Δd| (%)	I. D. Phase (R. Int.)
4.21	Strong										**4.14**	**100**	**1.7**							**Fe_2Ti (6th)**
3.48	Very strong				**3.32**	**210**	**4.8**													**OI-TiO_2 (4th)**
3.13	Strong	2.76	104	13.4	**3.15**	**111**	**0.6**													**OI-TiO_2 (3rd)**
																	2.71	110		
2.48	Weak	2.54	110	2.4				**2.49**	**111**	**0.4**							2.63	023	5.7	**FeO (2nd)**
											2.39	110								
					2.26	102		2.16	200		2.20	103								
					2.15	021					2.04	112								
					2.10	121														
2.02	Weak													**2.03**	**110**	**4.0**	**2.03**	**131**	**1.5**	**Fe (1st)** **Fe_2TiO_4 (4th)**
1.90	Weak	**1.87**	**024**	**1.6**							2.00	201	5.0							**$FeTiO_3$ (5th)**
1.71	Very weak	**1.73**	**116**	**1.2**							1.83	202	6.6							**$FeTiO_3$ (3rd)**
1.51	Strong	**1.51**	**214**	**0.3**				1.52	220	0.7										**$FeTiO_3$ (6th)**

Table S2 | Interplanar spacing measured from high-resolution image of Fig. 2a (HR3) and spacings of trigonal $FeTiO_3$, orthorhombic intermediate(OI) TiO_2 and orthorhombic Fe_2TiO_4. The spacing |Δd| is the difference between the d-spacing observed and that recorded in the database, I. D. Phase is the product phase identified by considering |Δd|, and R. Int. corresponds to the relative peak intensity in the database.

d_{obs} (Å)	Apparent Intensity	d_{hkl} $FeTiO_3$ (Å)	*hkl*	|Δd| (%)	d_{hkl} OI-TiO_2 (Å)	*hkl*	|Δd| (%)	d_{hkl} Fe_2TiO_4 (Å)	*hkl*	|Δd| (%)	I. D. Phase (R. Int.)
3.67		**3.74**	**012**	**1.9**	3.32	210	10.5				**$FeTiO_3$ (4th)**
2.98					**3.15**	**111**	**5.4**				**OI-TiO_2 (3rd)**
2.70		2.76	104	2.2				**2.71**	**110**	**0.4**	**Fe_2TiO_4 (1st)**
